# The Application of Whole Cell-Based Biosensors for Use in Environmental Analysis and in Medical Diagnostics

**DOI:** 10.3390/s17071623

**Published:** 2017-07-13

**Authors:** Qingyuan Gui, Tom Lawson, Suyan Shan, Lu Yan, Yong Liu

**Affiliations:** 1 Laboratory of Nanoscale Biosensing and Bioimaging, Instiute of Advanced Materials for Nano-Bio Applications, School of Ophthalmology and Optometry, Wenzhou Medical University, 270 Xueyuanxi Road, Wenzhou 325027, China; guiqyu@126.com (Q.G.); shan_suyan@163.com (S.S.); yanlu2017@wmu.edu.cn (L.Y.); 2 ARC Center of Excellence for Nanoscale BioPhotonics, Macquarie University, Sydney, NSW 2109, Australia; tomxlawson@gmail.com

**Keywords:** whole cell biosensors, bacteria, living cells, detection, bioluminescence, sensors, reporters, environmental analysis, diagnosis

## Abstract

Various whole cell-based biosensors have been reported in the literature for the last 20 years and these reports have shown great potential for their use in the areas of pollution detection in environmental and in biomedical diagnostics. Unlike other reviews of this growing field, this mini-review argues that: (1) the selection of reporter genes and their regulatory proteins are directly linked to the performance of celllular biosensors; (2) broad enhancements in microelectronics and information technologies have also led to improvements in the performance of these sensors; (3) their future potential is most apparent in their use in the areas of medical diagnostics and in environmental monitoring; and (4) currently the most promising work is focused on the better integration of cellular sensors with nano and micro scaled integrated chips. With better integration it may become practical to see these cells used as (5) real-time portable devices for diagnostics at the bedside and for remote environmental toxin detection and this in situ application will make the technology commonplace and thus as unremarkable as other ubiquitous technologies.

## 1. Introduction

A biosensor is a type of sensor that can detect and identify a component within a cell or tissue. It is composed of synthetically made biomolecule recognition elements and various kinds of physical or chemical transducers [1]. Because of the cross-disciplinary nature of their construction, research on their development has been published in the fields of biology, chemistry, physics, and information science [2,3,4,5]. These biosensors can be classified into three types based on differences in their molecular, cellular and tissue sensing components [6]. The molecular-based biosensors use biological active substances such as enzymes, DNA, antigens, antibodies, and biofilms as the reporter elements [7]. The significant advantage of these molecular-based biosensors is their high selectivity [8]. However, the applicability of this type of biosensor has been limited by shortcomings such as expensive macromolecule isolation costs, limited detection capability and the short useable lifetime of the identifying molecules [1].

In contrast to molecular-based biosensors, sensors that are formed from cells or intact tisse have seen rapid development in novel methods of their microfabrication and immobilization, and these quite recent changes have provided these types of biosensors with unique and unexploited advantages [9]. Though whole cell-based biosensors are not as sensitive to environmental changes as molecular-based ones, these biosensors can be modified by simple genetic engineering methods so that they can then be used to detect a series of complex responses within a living cell. These biosensors can further provide information that molecule-based biosensors are not capable of, such as information related to the pharmacology, cell physiology, toxicology of a sample.

**Figure 1:**
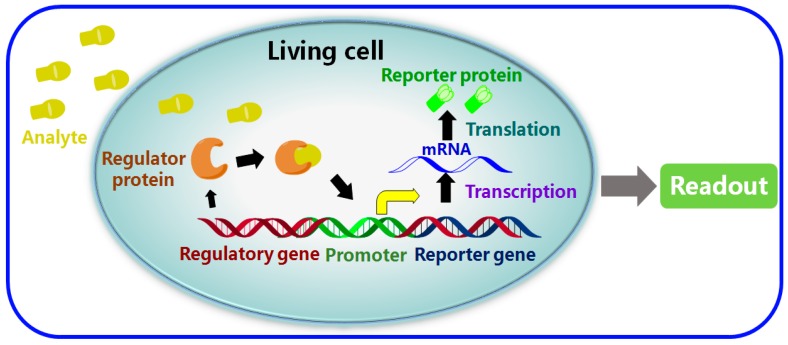
A schematic diagram representing a typical whole cell-based biosensor.

The main issues regarding the performance of a whole cell-based biosensor are: (1) the selection of reporter gene, as well as (2) the selectivity and sensitivity of the molecular recognition that occurs when regulator proteins bind to their target analytes [10]. As shown in Figure 1, the principal mechanism of a typical whole cell-based biosensor is the detection of a particular species of analyte and the amplification of this identification into an electrical and optical signal via a processor. This readout process is detectable through the immobilization and use of living cells or bacteria as the unit that provides the molecular recognition elements. Unlike a conventional biosensor, whole cell-based biosensors can detect a wider range of substances and thus are more sensitive to change in the electrochemical state of a tissue sample, other cells or in the environment, since they are capable of genetic modification and can operate over a broader range of conditions such as various temperature and pH values [11,12,13]. Because of the obvious advantages of whole cell-based biosensors such as their good sensitivity, high selectivity and their capability for high-throughput in situ detection, they have been applied successfully to fields such as environmental monitoring, food analysis, pharmacology and drug screening [10,14].

Pollutants, such as heavy metals and biocides, are now commonly found in places where there is rapid industrial transformation and these pollutants are considered a global threat [15]. Toxins in the environment typically are associated with detrimental health outcomes and loss of ecological diversity [16,17]. Fast and accurate detection of pollutants is essential to reduce these threats. Although conventional detection techniques for environmental pollution using physical chemistry methods show a certain degree of sensitivity and specificity, there are still many challenges that limit their practical application such as the need to use expensive equipment, complicated and involved procedures, and long delays for detection to be complete [18,19,20]. Conventional approaches also typically over-evaluate the total amount of environmental pollutants and usually cannot measure the bioavailability of the contaminants themselves [21]. Measurements associated with bioavailability are better able to indicate the toxicity and effects of pollutants on living organisms and people. Since Sanseverino et al. reported on the design of a whole cell-based biosensor for naphthalene detection in 1990, it has become commonplace for whole cell-based biosensors to be used for bioavailability detection and toxicity assessment of contaminants [7,22], as these biosensors have been shown to be economic, simple, and efficient for the in situ detection of the bioavailability of a broad range of environmental pollutants.

On the other hand, development of biomedical sensor technologies at a cellular and molecular level has become important to clinical diagnostics especially where it is used for the detection of chronic pathologies. Cells consist of naturally evolved receptor, ion-channels, and enzymes that can be used as targets for biological or biologically active analytes. Thus, whole cell-based biosensors are able to measure functional information and the effects of the analyte on the physiological function of living cells. Parameters can include determining the impact of a compound drug composition on the physiological system, the inhibition or promotion effect relative to a given receptor, other influences that the analyte might have on the metabolism of cells (such as secondary messengers and their enzymes), and the toxicity and the side effects of the substances tested have on cells. Whole cell-based biosensors have therefore emerged as a dynamic technique for qualitative and quantitative analysis of different analytes for clinical diagnosis [23].

In this short-review article, we summarize what has occurred recently in the design and development of whole cell-based biosensors with an emphasis on their application for environmental pollution monitoring and biomedical diagnostics, two areas where these sensors are most commonly applied. We provide a literature summary on the selection of reporter genes, regulatory proteins, host cells, and multifunctionalization, for the detection of environmental pollutants such as heavy metals and organic waste. We also provide an outline of recent developments in the application of whole cell-based biosensors to the field of precision medicine, detection of micronutrients and the diagnosis of diseases. This work further provides a summary of the potential challenges and future prospects of the practical application of whole cell-based biosensors.

## 2. Using Cells That Can Act as Sensors Against Environmental Analytes

### 2.1. Detection of Bioavailability

The rapid detection of pollutants in an ecosystem and the evaluation of their impact on health is an important focus for research. Conventional physical and chemical-based analytical methods can be highly accurate and sensitive in determining the exact composition and content of toxins in samples, but only a small number of toxins can be tested for their bioavailability, toxicity and genotoxicity [24]. In most cases it becomes only possible for some important parameters to be measurable by using living cells [25]. Two unique advantages of whole cell-based biosensors are: (1) the ease with which they can be field-tested and (2) the ease with which they can identify those fractions that contain a bioavailable contaminant. 

**Figure 2:**
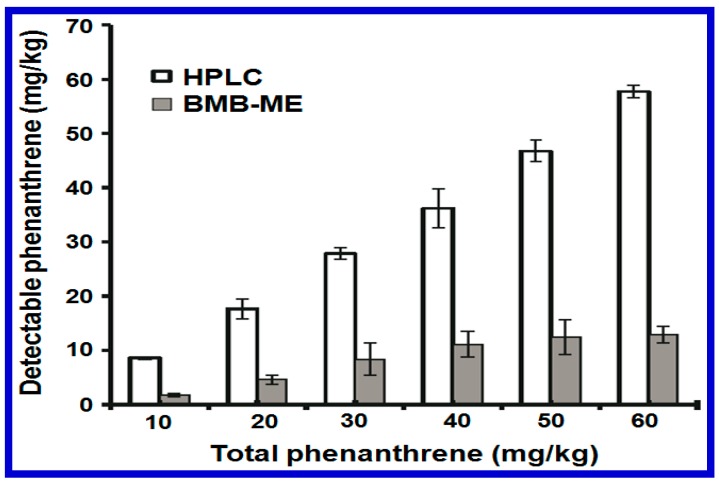
A comparison of the PHE concentrations detected using a HPLC or using a BMB-PL whole cell biosensor approach is given. The values represent the mean ± standard error (*n* = 3) for the fluorescence and bioluminescence detected. Reprinted with permission from [26].

An example of this is given in Figure 2, where a high-performance liquid chromatography (HPLC) and a whole cell-based biosensor Pseudomonas putida (BMB-PL) were tested and their ability to detect phenanthrene (PHE) added to red soil samples was compared [26]. The initial concentration of PHE was tested at various values from 10 to 60 mg/kg. It was found that around 80% PHE was detected by HPLC. In contrast, the application of BMB-PL was able to detect the bioavailable fraction at levels much lower than those tested with the total amount of PHE. This was mainly due to the sample extraction procedure during HPLC measurement. Besides the detection of organics, Pasi et al. also tested the bioavailability of Pb and Cu concentrations in natural soil using a similar bioluminescent whole cell biosensor. This result was again consistent with the results demonstrated by the PHE study; a much higher selectivity was obtained using the whole cell-based biosensor [27]. The chemical analysis was also found to be more expensive, time-consuming, and required a specialized laboratory to perform its procedure. Finally, whole cell-based biosensors were shown to be able to continually monitor the bioavailability and concentration of toxic compounds in situ to the sample and and in real time [28].

### 2.2. Reporter Genes

Typically, the performance of whole cell biosensors for the detection of environment pollutants is dependent on the reporter genes chosen for the control of transcriptional contaminants, and the type of regulatory protein associated with these promoters. A reporter gene in living cells used as sensors can convert its biological response into a signal detectable physicochemically. This process is critical to the sensitivity and selectivity of the whole cell biosensors. There are some widely used reporter genes that were shown to incorporate successfully into whole cell-based biosensors. These include lux (Bacterial lucberase), luc (firefly luciferase), gfp (green fluorescent protein) and lacZ (β-galactosidase). The selection of which reporter gene to use can be difficult since there is a large number from which to choose from. Table 1 lists some of the advantages and disadvantages of commonly reported reporter genes used to form a whole cell-based biosensor.

Gfp is a reporter gene coding for the green fluorescent protein (GFP) and it autofluorescences, so it does not require a substrate or an ATP to emit [34]. But with this reporter, the intrinsic fluorescence of certain host cells generally increases the background fluorescence and this can cause signal interference. So gfp-based biosensors are usually not able to detect with as much sensitivity as other lux- and lacZ based biosensors [35]. In addition, GFP requires a longer time to emit a stable fluorescence, reducing its maximum detection activity [36]. Thus, gfp-based whole cell biosensors are typically unsuitable for the rapid detection of containments. Likewise, the bacterial luciferases (lux) generally suffer from thermal lability and dimeric protein interference, and this also limits its use as a reporter gene in mammalian cells [37]. To avoid these limitations, the firefly luciferase (luc) reporter was frequently incorporated into mammalian cells considering its high sensitivity and broad linear range (up to 7–8 orders of magnitude) [38,39]. Another reporter, β-Galactosidase (lacZ), a well-characterized bacterial enzyme, was also frequently used in molecular biology as it is an excellent monitor of transfection efficiency. lacZ exhibits some unique advantages for detection using either colorimeric or fluorescent methods as its use with a sample is simple and rapid [40]. The wide availability of chemiluminescent and electrochemical substrates for lacZ also provides the advantages of ultra-high sensitivity, low detection limit (as low as 2 fg) and an extensive dynamic detection range [41,42].

In another reporter system, Fujimoto and coworkers developed a novel type reporter gene, crtA that is responsible for carotenoid synthesis [43]. When applied to a sample, the crtA-based whole cell-based biosensors change the color of the culture media from yellow to red without the addition of a supporting substrate, and is thus considered a good choice for rapid detection in emergency situations [44]. Table 2 compares the biosensing sensitivity of recently developed whole cell-based biosensors for the detection of environmental pollutants such as heavy metals and organic waste. A variety of cell lines and reporter genes were used in these systems. For instance, Sharma et al. utilized luxCDABE and *E. coli* to design whole cell-based biosensors and these were demonstrated to have a detection sensitivity of 0.74 μg/L when arsenic was added to water, a level well below the minimum safety standards for arsenic (10 μg/L) in the EU and in the United States [45]. In addition, the detection sensitivity of whole cell-based biosensors to organic waste was found to be excellent and showed great potential.

### 2.3. Regulatory Proteins

**Figure 3:**
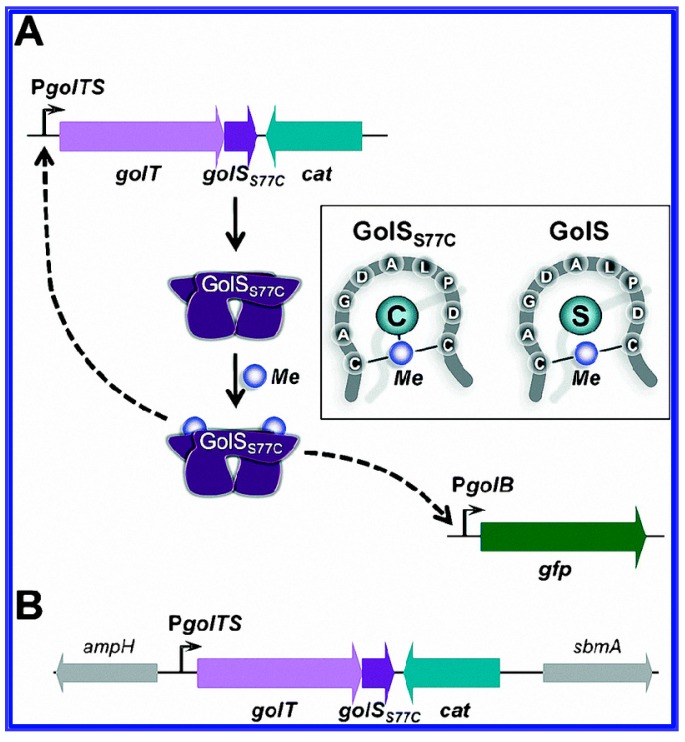
(**A**) The GolS77C-based biosensor platform is shown. The sensor protein GolSS77C is expressed using its chromosomally encoded gene with an operon with golT encoding the P1B-type Au (I) transporter (STM14_0413-STM14_0412); (**B**) Genetic organization of the site chosen for the insertion of the golTSS77C-cat locus in the *E. coli* chromosome is also given. Reprinted with permission from [57].

The regulatory protein, which possesses complex interactions with the target analytes of the contaminants of interest, is essential for the specificity and sensitivity of the whole cell-based biosensors. In recent years, with the discovery of metallo-regulatory proteins, there are many reports elsewhere that utilized these biosensors for the detection of heavy metals in water and soil samples. These have shown higher selectivity, expanded detection ranges and enhanced sensitivity when compared to conventional biosensors. For example, GolS protein, a regulatory protein of the MerR family, was reported having a high selectivity for Au ions [56]. It was also found that with the introduction of a single amino acid at the position 77 in the GolS protein [57], the detection ranges of GolS-based whole cell biosensors were improved and became suitable for the detection of mercury (Hg), lead (Pd), cadmium (Cd) and/or gold (Au) ions (Figure 3). Metallothionein (MT), a cysteine-rich peptide, was reported having a high affinity to various heavy metals, and consisted of five isoforms encoded by *T. thermophila* genes [58,59]. In a report by Amro et al., a whole cell biosensor with MTT5 and MTT1 promoters (separated from MTs) was constructed, and it responded rapidly and strongly to the presence of heavy metal contaminants [10]. Whangsuk et al. discovered that the *Sinorhizobium meliloti* chpA promoter was highly induced by the pesticide chlorpyrifos (CPF) through the inclusion of the transcriptional activator ChpR [60].

In another report, the biosensor with a chpA promoter was hosted in *Escherichia coli* and used for the detection of CPF over a linear response range of 25 to 500 nM CPF [61]. Elsewhere, Fujimoto et al. developed a highly sensitive whole cell biosensor consisting of the promoter region of the ars operon and a reporter gene, the crtA gene, and were able to efficiently detect arsenite [43]. The color change was clearly recognized by the naked eye, even when the concentration of arsenite present was at 5 μg/L. The ars operon was also reported having an association with arsenite resistance, while the crtA gene, which determines the carotenoid synthesis in *Rhodovulum sulfidophilum*, was responsible for the color of the culture changing from yellow to red as the concentration of arsenite changed [44,62]. 

DNA microarrays are a technology that can be used as a high-throughput method for biosensors to select for positive regulatory genes as these arrays contain many protein, one of which typically responds to presence of the pollutant of interest in a sample. For example, Byoung et al. utilized DNA microarray data to select appropriate biomarker genes that were highly induced after exposure to the toxin paraquat [63]. Through two rounds of directed evolution of the transcriptional regulator, AlkSp protein, an alkane-inducible biosensor for detection of short-chain alkanes was constructed and the authors were able to demonstrate a 5-fold emission increase in its reporter signal [64].

### 2.4. Host Cells

**Figure 4:**
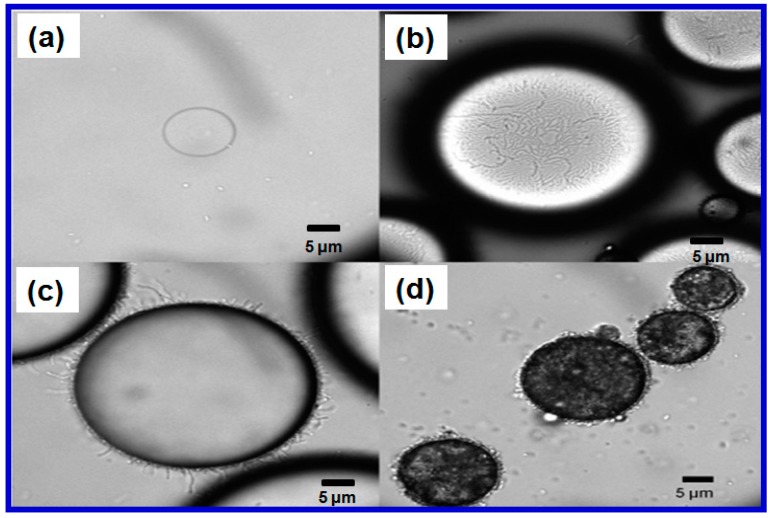
(**a**) Poor accessibility and emulsifying capability of *E. coli* (the circle) towards oil droplets. Good affinity and emulsifying capability of acinetobacter baylyi ADPWH-alk (the circles) towards the surface of (**b**,**c**) the mineral oil droplets; and (**d**) the curde oil droplets. Reprinted with permission from [67].

The selection of the type of host cell is also important. A biosensor’s specificity, sensitivity and time-response, can be greatly influenced by the type of host cells used as the vehicle for sensing. Since there is a high similarity in metabolism, genome, and cellular organization between eukaryotic-based biosensors and a host organism, about 85% of whole cell biosensors currently used for detection of metals are eukaryotes [65]. Hernández-Sánchez and their coworkers constructed different whole cell biosensors using various host cells, but the same recombinant regulatory system, for the detection of monocyclic aromatic compounds in different environmental samples [66]. The biosensor from *Alcanivorax borkumensis* SK2 was reported as having a lower tolerance towards pollutants, but a higher tolerance towards salinity, and it demonstrated the best performance for detecting pollutants at low concentrations in seawater samples. The biosensor hosted on *Pseudomonas putida* DOT-T1E was found to be the best option for heavily contaminated environments due to its highest solvent tolerance at high concentrations before it bacame saturated [64]. As shown in Figure 4a, it was hard to obtain small oil droplets in the *E. coli*-oil mixture due to the poor accessibility and emulsifying capability of *E. coli* (the circle in Figure 4a) towards oil droplets. However, *Acinetobacter baylyi* ADP1 and its derivative ADPWH-alk (the circles in Figure 4b–d) were naturally adhesive to an oil-water interface and can emulsify both mineral and crude oils into oil droplets that are about 10–80 μm in diameter (Figure 4b–d) [67]. These properties make ADP1 an excellent bacterial substrate for constructing whole cell biosensors for detecting a broad range of alkanes and alkenes with carbon chain lengths from C_7_ to C_36_ found in samples such as water, seawater and soils [67,68,69].

A biosensor based on *Alcanivorax borkumensis*, specialized in assimilating linear alkanes, and exhibited a four-fold lower detection sensitivity towards the fuel octane (0.5 μM) when compared to biosensors that used *Escherichia coli* as a vehicle. This improvement in performance was most evident when measuring low concentrations of pure alkanes or petrol in samples [70]. Brutesco et al. prepared a functional biosensor based on *Deinococcus deseri* (an environmental, desiccation- and radiation-tolerant bacterium), and reported that these sensors were able to detect arsenite after 7 days of storage [71]. A series of whole cell biosensors were also prepared from several *Escherichia coli* strains for the detection of nickel in drinking water. In a typical example, the TD2158 wild-type *E. coli* showed a 10 times higher activity and sensitivity than its W3110 *E. coli* K12 equivalent, even though the same mechanism was used with RcnR Ni/Co metallo-regulator and a rcnA natural target promoter fused to the luc reporter genes was used [72]. In this work, the detection limit for Ni was reported to be as low as 80 nM, and so was found useful for meeting the required quality standards for most drinking water. These studies demonstrated that the choice of host bacteria has a significant impact on the performance of a fully constructed whole cell-based biosensor.

### 2.5. Multifunctionalization

**Figure 5:**
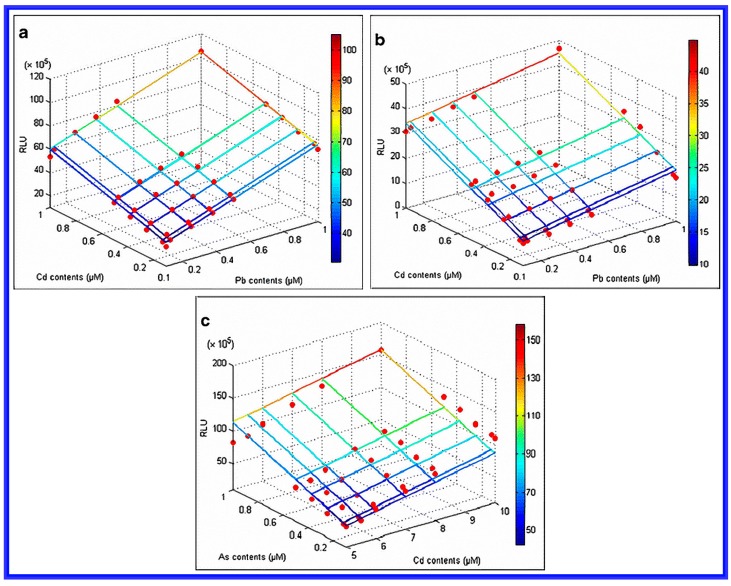
Binary linear regressions of (**a**) a pcdCluc sensor in Group 1 to Cd-Pb mixtures; (**b**) a pzntRluc sensor in Group 1 to Cd-Pb mixtures; and (**c**) a parsRluc sensor in Group 2 to As-Cd mixtures are given. Reprinted with permission from [77].

Although whole cell biosensors typically exhibit better sensing performance than conventional chemical-based biosensors, the literature on the subject that has had a continual focus on improving the sensitivity, accuracy, and applicability of these sensors. This concern possibly reflects an understandable reaction to the widespread and increasing levels of pollution [73]. Recently, different types of functional whole cell biosensors were mixed together and were shown to be better at contaminant detection and measurement than a single biosensor type. For example, a combination of bioluminescent bacteria-based toxicity screening and a yeast-based estrogenic activity assay was applied to the monitoring of water samples [74]. Here, multiple biosensors were prepared using a combination of regulatory proteins such as CadC, ZntR, and ArsR, which then simultaneously responded when several different metals were added [75,76]. In another example, for the purpose of improving the specific and accurate detection of bioavailable Cd, Pb and As in a co-contaminated environment, a tailored whole cell biosensor set (Figure 5) was prepared by analyzing a *E. coli* sensor set using binary regression and binary linear equations to minimize the interference in signal generated when different metals (such as Cd, Pb and As) were presented [77].

In that work [77], sensor sets were classified into two groups according to their specific response to Cd, Pb and As. Group 1 was composed of pcadCluc and pzntRluc sensors to identify the bioavailability of Cd and Pb; Group 2 consisted of a parsRluc sensor to detect the specific bioavailable As. The increased concentration range of mixtures resulted in a linearly increase in the relative light unit (RLU). Three binary liner equations for two sensor groups were then used to determine the concentrations of bioavailable Cd, Pb, and As in soils sampled from a co-contaminated mine site. With Group 1, the overlapping concentration ranges (0.1–1.0 μM) of mixed Cd-Pt were determined using a linearly increased RLU. For Group 2, the overlapping concentration ranges were found to be 0.1–1.0 μM for As, and 5.0–10.0 μM for Cd. These results in this article showed that a conventional single target whole-cell sensor system tended to overestimate the bioavailability of the heavy metals detected. More accurate bioavailability data were obtained when a multiple whole cell biosensor were applied.

This review was, in part, born from increasing concerns with the so-called “antibiotics crisis” which relates to the excessive and unnecessary pesticides, antimicrobials use, and their uncontrolled environmental release [78]. There is an urgent demand for the development of whole cell-based biosensors for the stable and rapid detection of diverse antibiotics in samples sourced from extremely polluted environment. For example, microbial biosensors based on *Pseudomonas putida* DOT-T1E have shown a capability to detect a wide range of structurally diverse antibiotics due to its adaptability in the environmental and its resistance to a diverse number of toxic organic compounds [79].

Though whole cell-based biosensors have typically exhibited superior performance compared to their conventional chemical counterparts, their commercialize realization is still a major challenge. Reproducibility and stability during long-term storage and transport is still not efficient enough to meet the demands for their large-scale fabrication. Camanzi and colleagues, however found that freeze-dried bacteria emitted a stable luminous signal after their rehydration several months later [80]. Prévéral et al. developed an arsenite based whole cell biosensor that still retained its performance for the sensitive detection of arsenite seven months' after its lyophilization [81]. In that report, the entire bacterial luciferase lux operon (luxCDABE) with autonomous bioluminescence emission was used as the reporter gene, while lux a constitutive *E. coli* promoter PrpoD drove its expression. In another report, a flow-through biosensor based on disposable modular biochips incorporating agar-immobilized bioluminescent recombinant reporter bacteria, was proposed for the online and continuous monitoring of water toxicity [82]. This whole-cell biosensor was shown to work well in continuous flowing water over several days.

## 3. The Use of Sensors Based on Whole Cells in Medical Diagnostics

Cells possess and express a series of molecular recognition elements, such as receptors, ion channels, and enzymes. These molecules are usually sensitive to their corresponding analytes because of their native cellular mechanism [83]. Whole cell-based biosensors can therefore be used to continuously monitor and analyze a variety of physiological parameters under an external stimulation such as changes to the cell’s metabolism, impedance and action potential. These living cell-based sensors can thus be used to understand biological metabolic disorders and other diseases at a cellular level, and this has lead to these biosensors becoming widely applicable to many fields in biomedicine such as cellular physiological analysis, pharmaceutical evaluation and medical diagnosis.

### 3.1. Precision Medicine

A current research aim of precision medicine is to explain the genetic mutation of drug targets such as G protein-coupled receptors (GPCRs) and changes to their response to drugs . For instance, a novel label-free, whole cell-based biosensor was designed to characterize GPCR-mediated drug responses in lymphoblastoid cell lines (LCLs) [84]. This suggests their potential use as a cellular model system for the investigation of GPCR pharmacology in vitro in precision medicine. For example, Feng et al. fabricated a whole cell biosensor using a bullfrog fibroblast cell line (FT cells) expressing GPCRs as its basis, to evaluate the activity of GPCRs and determine the amount of adrenaline they secreted [85]. Here, the tumor suppressor gene p21 was used because it is a dominant downstream target gene of the activated p53 protein and is susceptible to carcinogens and can thus act as a sensor. In another report, Zager and co workers tested a human hepatoma cell-based biosensor that used a plasmid encoding enhanced green fluorescent protein (EGFP) under the control of a p21 promoter, for the simple and fast detection of genotoxic agents [86].

### 3.2. Detection of Micronutrients

**Figure 6:**
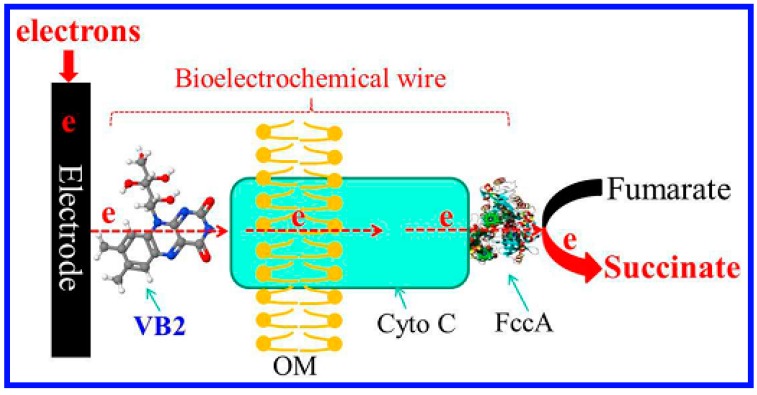
A schematic diagram illustrating the electron transfer pathway used in bioelectrochemically-based whole cell biosensors. OM indicates the outer cell membrane; Cyto C represents cytochrome C proteins; and FccA indicates fumarate reductase. Reprinted with permission from [91].

Another area of application for whole cell sensors is in field of micronutrients. Riboflavin is an essential vitamin for human health and its lack of it can lead to serious diseases such as metabolism disorders, cataracts, and some cancers [87,88]. Excess intake of riboflavin can also be harmful and lead to oxidative damage in light-exposed tissue [89,90]. To address this, Si et al. reported a whole cell bioelectrochemical biosensor system for the amperometric detection of riboflavin [91]. As shown in Figure 6, a bioelectrochemical wire (BW) was made and consisted of riboflavin and cytochrome C strung between *Shewanella oneidensis* MR-1. An electrochemical response was obtained when riboflavin was added to the BW system. In comparison to conventional chemical biosensors, a 200 times enhancement in the electrochemical signal output was observed. The as-prepared whole cell biosensor had a high sensitivity (2.2 nM, S/N = 3), a wide linear range (5 nM–10 μM, 3 orders of magnitude) and a high resistance to signal interference.

### 3.3. Diagnosis of Diseases

**Figure 7:**
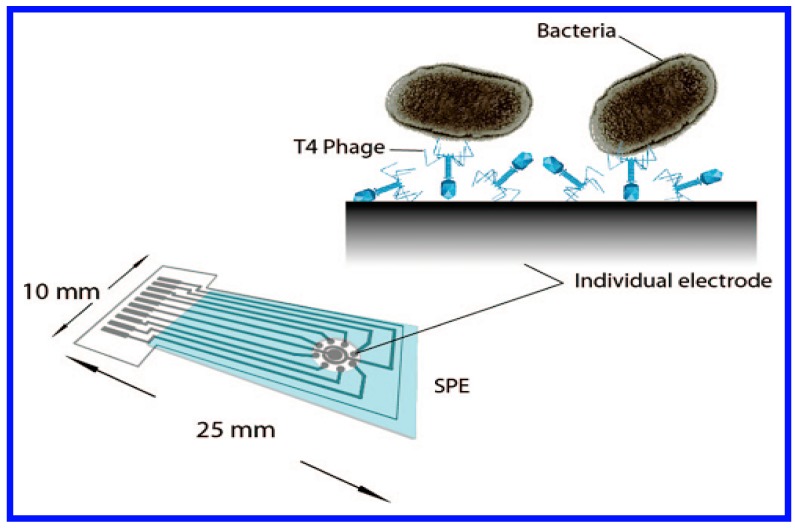
A schematic diagram of the assay for immobilizing phages onto electrochemical electrodes is shown. Reprinted with permission from [92].

Rapid and accurate detection of pathogens is an important goal in the diagnosis of diseases. Conventional microbiological methods can require several days to weeks since microbes in patient blood and urine samples have to first be precultured to sufficient numbers for their detection. A novel technique based on the whole cell biosensors was designed for the specific and direct detection of bacteria that did not require this lengthy culture step [92]. As seen in Figure 7, the whole cell biosensor, that included bacteriophages acting as recognition receptors, was immobilized covalently to a functionalized screen-printed carbon electrode (SPE) microarray for the direct impedimetric detection of *E. coli*. The results of the impedance measurement indicated that the biosensor acted as a rapid, direct, and label-free method for detecting specific bacteria, at the low cell numbers typically seen in patient samples.

Whole cell-based biosensors can provide high-content screening and analysis in the diagnosis of diseases. For example, bacteria quorum-sensing molecules (QSMs) influenced 4% to 10% of bacterial genome and more than 20% of its proteome, indicating that quorum sensing was associated with both modulate virulence factor production and the basic metabolic processes [93]. Whole cell-based biosensors have also been used as the non-invasive methods to evaluate QSMs in physiological samples sourced from patients affected by bacterial gastrointestinal disorders [94,95]. The detection limits of QSMs were improved to the nanomolar level in biological matrices [96]. This was important, as invasive non-typhoid *Salmonella* (iNTS) is a main cause of human mortality and morbidity seen in young children and at-risk adults in sub-Saharan Africa [97]. Venkatesh et al. also reported a whole cell based electrochemical immunosensor, hosted in yeast, to detect iNTS antigens [98]. In that report, yeast cells were genetically modified to display both single chain variable fragment (scFv) antibodies and gold-binding peptide (GBP) on their surfaces. The resulting whole cell sensor showed a wide dynamic range with high nanomolar sensitivity and was capable of detecting iNTS OmpD antigens [98].

The unique advantages of whole cell-based biosensors have arisen in part because of their simplicity and fast application to result time for diagnosis of various diseases. Whole cell biosensor based biomedical diagnosis techniques thus show promise and great potential in the area of biomedical engineering.

## 4. A Summary on the Use of Whole Cell-Based Biosensors and Their Prospect

Biosensors formed from living cells have become an attractive field for researchers because of the rapid pace of innovations seen in microfabrication and in cell immobilization. Whole cell-based biosensors can quantitatively detect information relating to the status of live animal or bacteria cells, by converting signals that form part of their homeostasis, into electrical and optical outputs that can then be detected. Compared to conventional chemically based biosensors, these whole-cells can provide real-time, rapid and unique data streams of the homeostatic status of the cell and by inference, and its microenvironment, to a very high sensitivity and selectivity. This work reviewed recent progress in development and use of whole cell-based biosensors and their application across a range of fields with a particular focus on the area of environmental pollution monitoring and medical diagnostics. 

Whole-cell based biosensors have many advantages, but there are also limitations to their use. Most environmental samples tested contain a large number of pollutants as well as other naturally occurring molecules and together these mask the signal coming from the analyte of interest [99]. Another issue is the toxic nature of the samples, as these can contain heavy metals or organic pollutants and their presence can limit the choice of cells used to those microbes resistant to their action. Finally, emission from whole cell-based sensors employed for extended periods of time can become unstable over time as these cells undergo leakage or diffusion. One solution reported elsewhere was to first immobilize cells onto a substrate before their application to a sample for testing [100].

The future prospects of whole cell-based biosensors may prove exciting. An early example of what this future might hold was demonstrated in a report on a portable whole cell-based optical biosensor named Luisens 2 developed to provide real-time online detection of pollutants [101]. The biosensor consisted of three main parts: (1) a central unit; (2) a disposable card where the bacteria were first immobilized and inserted; and (3) an acquisition unit to control the device. All kinds of recombinant bioluminescent bacteria were immobilized onto the disposable card. In another example, a portable whole cell biosensor system for environmental monitoring was demonstrated [102]. This system consisted of a lightweight mechanical housing, a temperature regulation system and a microfluidic housing bacterial inoculation channel. The system was used as a portable incubator for cell inoculation and as a remote monitor for testing at unreachable locations by its attachment to an unmanned aerial vehicle.

Another future focus for whole cell-based biosensors is the fabrication of specific and multifunctional biosensors for rapid and real-time detection in extreme unfriendly environments where there is high acidity, alkalinity, high salinity, extreme temperature, and toxic substrates [103]. It could be envisaged that many cell types, such as thermophiles, alkalophiles, halophiles and etc, could be applied as host-cells for their use as whole cell-based biosensors for extreme environmental analysis.

## Figures and Tables

**Table 1:** A summary of typical reporter genes used in whole cell-based sensors.

Gene	Detection Method	Advantages	Disadvantages
lux [29]	Bioluminescence	Easy measurement, rapid response	Thermal lability, O_2_ requirement
luc [30]	Bioluminescence	High sensitivity, rapid response, thermal stability	O_2_ and ATP requirements, low permeability
gfp [31]	Fluorescence	No substrate requirement, high stability	Low sensitivity, lag-time for stable fluorescence, autofluorescence
lacZ [32]	Bioluminescence, Fluorescence, Colorimetry, Electrochemistry	High stability, wide variety of detection methods, detection by naked eyes	Substrate dependent, low permeability
crtA [33]	Colorimetry	Detection by naked eyes	Substrate dependent

**Table 2:** A comparison of the sensitivities for different types of whole cell-based biosensors.

Host Chassis	Reporter Gene	Target Analyte	Detection Sensitivity	Reference
*E. coli*	luxCDABE	arsenic	0.74–69 μg/L	[45]
*E. coli*	lacZ	arsentate	<10 μg/L	[46]
*D. radiodurans*	lacZ	cadmium	1–10 mM	[47]
	crtI		50 nM–1 mM	
*E. coli*	Gap	chromate	100 nM	[48]
*E. coli*	Gfp	zinc	16 μM	[49]
		copper	26 μM	
*Escherichia coli*	Luc	benzene, toluene and xylene	40 μM	[50]
*E. coli*	luxAB	benzene, toluene and xylene	0.24 μM	[51]
*P. putida*	luxAB	phenol	3 μM	[52]
*B. sartisoli*	luxAB	naphthalene and phenanthrene	0.17 μM	[51]
*E. coli*	luxAB	C_6_–C_10_ alkanes	10 nM	[53]
*E. coli*	luxCDABE	tetracyclines	45 nM	[54]
*S. typhimurium*	lacZ	single-stranded DNA	10 nM mitomycinC	[55]
